# 12-month prevalence of osteoarthritis in Germany

**DOI:** 10.17886/RKI-GBE-2017-066

**Published:** 2017-10-09

**Authors:** Judith Fuchs, Ronny Kuhnert, Christa Scheidt-Nave

**Affiliations:** Robert Koch Institute, Department of Epidemiology and Health Monitoring, Berlin

**Keywords:** OSTEOARTHRITIS, PREVALENCE, ADULTS, HEALTH MONITORING, GERMANY

## Abstract

Osteoarthritis is the most common joint disease worldwide. In the advanced stage the disease is characterised by joint pain and loss of joint functionality. In the Robert Koch Institute’s GEDA 2014/2015-EHIS health interview survey, 17.9% of adults over 18 reported having suffered from osteoarthritis during the past twelve months, whereby prevalence for women (21.8%) was higher than for men (13.9%). Osteoarthritis becomes more common with age. Among those aged 65 and over, around half of all women (48.1%) and nearly one third of men (31.2%) are affected. Due to population ageing, the prevalence of osteoarthritis in Germany can be expected to increase further in the future.

## Introduction

In Germany as well as globally, osteoarthritis is the most common joint disease [1, 2]. The disease is characterised by degenerative processes in the joints that begin with the gradual erosion of joint cartilage, and which can lead to the full exposure of bone surfaces. The bones, muscles and ligaments attached to the affected joints can also be affected [3]. Non-modifiable risk factors for osteoarthritis include older age, female gender and genetic predisposition. Further acquired (or contributing) causes include overstress and misload of joints due to congenital misalignments (axis misalignment, hip dysplasia); injuries and accidents; excessive physical activity or inactivity and overweight or obesity. At advanced stages, osteoarthritis causes pain and leads to joint dysfunction, both of which result in loss of mobility, physical disability and limitations in usual activities, and consequently significant loss of quality of life. Beyond the personal burdens suffered by osteoarthritis patients, the disease also generates considerable economic costs [3].

Numerous efforts at the international level currently target musculoskeletal diseases and the related disease burden. When the World Health Organization (WHO) endorsed the Bone and Joint Decade 2000-2010, experts estimated that due to demographic developments the number of patients affected by bone and joint diseases would approximately double between 2000 and 2020 [2]. The WHO strategy therefore focussed on promoting research and enhancing the quality of healthcare services. This initiative led to an increased awareness for musculoskeletal diseases and the insight that the capacity to provide prompt and adequate care would require new models of care [4].

Given the ageing of society, osteoarthritis prevalence in Germany will continue to rise. This will lead to further burdens on the healthcare system. Considering the identified risk factors for osteoarthritis, which can be divided into those at the person level (age, sex, obesity, genetic predisposition, physical activity and diet) and those at the joint level (injuries, malposition and overstress) [5], sufficient evidence to justify interventions exists only for modifiable risk factors such as obesity and joint injuries [6].

**GEDA 2014/2015-EHIS**

**Data holder:** Robert Koch Institute

**Aims:** To provide reliable information about the population’s health status, health-related behaviour and health care in Germany, with the possibility of a European comparison

**Method:** Questionnaires completed on paper or online

**Population:** People aged 18 years and above with permanent residency in Germany

**Sampling:** Registry office sample; randomly selected individuals from 301 communities in Germany were invited to participate

**Participants:** 24,016 people (13,144 women; 10,872 men)

**Response rate:** 26.9%

**Study period:** November 2014 - July 2015

**Data protection:** This study was undertaken in strict accordance with the data protection regulations set out in the German Federal Data Protection Act and was approved by the German Federal Commissioner for Data Protection and Freedom of Information. Participation in the study was voluntary. The participants were fully informed about the study’s aims and content, and about data protection. All participants provided written informed consent.

More information in German is available at

www.geda-studie.de

## Indicator

The osteoarthritis indicator in the GEDA 2014/2015-EHIS health interview survey was calculated using a self-administered paper-based or online questionnaire. Numerous diseases and conditions were assessed using the questions: ‘During the past twelve months have you had any of the following diseases or conditions?’ and: ‘Has a physician ever diagnosed you with any of the following diseases or conditions?’, which was followed by a list of diseases. Participants were expected to provide information on whether they had had osteoarthritis during the past twelve months. The results are presented stratified according to gender, age group and educational level.

The data presented are based on participants’ self-assessment of twelve-month prevalence. We can assume these self-assessments to be reliable, but will expect them to lead to lower prevalence rates than for example radiological examinations would [7, 8].

The analyses are based on the data from 22,753 participants aged 18 years and older (12,481 women, 10,272 men) with valid data on osteoarthritis. The calculations were carried out using a weighting factor that corrects for deviations within the sample from the German population (as of 31 December 2014) with regard to gender, age, district type and education. The district type accounts for the degree of urbanisation and reflects the regional distribution in Germany. The International Standard Classification of Education (ISCED) was used to classify the responses provided on educational level [9].

For a detailed description of the methodology applied in the GEDA 2014/2015-EHIS study see Lange et al. 2017 [10] as well as the article German Health Update: New data for Germany and Europe, in Issue 1/2017 of the Journal of Health Monitoring.

## Results and discussion

17.9% of adults over 18 report having had osteoarthritis during the past twelve months, whereby prevalence among women (21.8%) is higher than among men (13.9%).

Osteoarthritis is more common among older than among young people. During early adulthood (18 to 29 years) osteoarthritis is very rare with a prevalence of 0.9% among women and 0.4% among men. Among 30-to 44-year-olds this rises to 4.3% (women) and 4.1% (men) (Table 1) and then increases steeply, with 23.2% of women and 16.6% of men in the 45 to 64 age group reporting cases of osteoarthritis. In the age group of those over 65 years of age nearly half of all women (48.1%) and a third of all men (31.2%) are affected. Women in the 45 to 64 and over-65 age groups are affected significantly more frequently by osteoarthritis than men.

In the 45 to 64 age group a correlation between osteoarthritis and education is apparent for both sexes. People with higher levels of education report osteoarthritis significantly less often than those from a medium- or low-education background. For the other age groups no significant differences by level of education were observed.

Stratified by federal state there are no regional differences in the prevalence of osteoarthritis.

Comparisons between these results and the findings of previous national health surveys regarding the prevalence of osteoarthritis in Germany are difficult: Methods regarding both the collection of data (telephone interview, paper-based questionnaire, interview by physician) as well as the operationalisation (how questions were posed) of indicators have changed over time, which can lead to discrepancies in prevalence estimates. Personal interaction between interviewers and interviewees and thus, possibilities to clarify questions, for example, can have an impact on responses. Similarly, the composition of a survey’s population sample (for example according to age, sex, social status, acute pain) can also lead to discrepancies in prevalence estimates between different surveys [11, 12]. In its 2012 interview survey (GEDA 2012) the Robert Koch Institute reported that 24.5% of women and 16.1% of men aged 18 and over had ever been diagnosed with osteoarthritis or another degenerative joint disease by a physician and that this osteoarthritis had persisted into the past twelve months. The available sex-stratified results on the 12-month prevalence of osteoarthritis from GEDA 2014/2015-EHIS are marginally lower, which might be due to the fact that the question here did not include degenerative joint disease. The German Health Interview and Examination Survey for Adults (DEGS1, 2008-2011) surveyed the lifetime prevalence of a medical diagnosis of osteoarthritis or other degenerative joint disease among adults aged 18- to 79-years based on personal interviews. Compared to current prevalence estimates, DEGS1 results for women (22.3%) did not differ much, whereas the estimates for men (18.1%) were significantly higher than in the current study [13]. Results on osteoarthritis prevalence among adults aged 18 to 79 years from an even earlier German national health survey are also not comparable to the current analysis, neither regarding how the question was posed (asking whether a physician had ever diagnosed osteoarthritis or another degenerative joint disease of the hips, knees or spine), nor regarding the timespan (ever diagnosed, persisted into the past four weeks). In this earlier analysis, the estimated overall prevalence was 27.7%. Significantly higher prevalence rates among women were only evident for the older age groups [14]. All publications consistently reveal a significant increase in osteoarthritis prevalence for the second half of life [3, 12, 13, 15].

GEDA 2014/2015-EHIS did not assess the localisation of osteoarthritis. DEGS1 results [11] reveal that in more than half of participants osteoarthritis affected the knee and in about one quarter the hip joint. Around one third of women and one seventh of men reported osteoarthritis in their finger joints. Half of those suffering osteoarthritis reported further joints were affected as well.

Overall, osteoarthritis is a common disease among older people in Germany. Joint pain and loss of function are likely to lead to a loss of quality of life. Osteoarthritis in the lower extremities (hips and knees) obviously involves a higher degree of loss of mobility than osteoarthritis in the upper extremities (fingers, hands and shoulders). Preventive healthcare strategies should first and foremost focus on preventing obesity and joint injuries. With patients who have already developed osteoarthritis the focus should be on maintaining joint functionality. Establishing a continuous database for analyses of time trends as well as collecting additional information through interview and examination surveys on comorbidity, loss of function, administered medications, joint replacements or joint pains will be key to describing the development of osteoarthritis (see the Fact sheet in this issue).

## Key statements

- Around 22% of women and 14% of men suffer from osteoarthritis during the past twelve months.
- Osteoarthritis rates increase with age and particularly during people’s second half of life. Nearly half of all women and one third of all men aged 65 and over suffer from osteoarthritis.
- Among persons in middle adulthood, osteoarthritis prevalence is lower among women and men with high education than among women and men with medium or low education.

## Figures and Tables

**Table 1:** 12-month prevalence of osteoarthritis stratified according to gender, age and educational level (n=12,481 women; n=10,272 men) Source: GEDA 2014/2015-EHIS

Women	%	(95% CI)	Men	%	(95% CI)
**Women total**	**21.8**	**(20.9-22.7)**	**Men total**	**13.9**	**(13.0-14.8)**
**18-29 Years**	0.9	(0.5-1.5)	**18-29 Years**	0.4	(0.2-1.2)
Low education	0.9	(0.2-3.4)	Low education	0.2	(0.0-1.4)
Medium education	0.9	(0.5-1.7)	Medium education	0.5	(0.1-2.1)
High education	0.9	(0.3-2.6)	High education	0.7	(0.2-2.2)
**30-44 Years**	4.3	(3.4-5.3)	**30-44 Years**	4.1	(3.2-5.3)
Low education	3.8	(2.0-7.1)	Low education	4.4	(1.9-9.9)
Medium education	5.1	(3.9-6.5)	Medium education	4.3	(3.0-6.0)
High education	2.6	(1.9-3.7)	High education	3.7	(2.5-5.5)
**45-64 Years**	23.2	(21.7-24.7)	**45-64 Years**	16.6	(15.2-18.2)
Low education	29.1	(24.9-33.6)	Low education	19.2	(15.1-24.0)
Medium education	23.1	(21.3-25.0)	Medium education	18.7	(16.5-21.2)
High education	18.0	(15.9-20.2)	High education	12.0	(10.3-13.9)
**≥ 65 Years**	48.1	(45.6-50.6)	**≥ 65 Years**	31.2	(28.9-33.7)
Low education	47.9	(44.0-51.7)	Low education	31.7	(27.0-36.8)
Medium education	48.1	(44.7-51.5)	Medium education	32.6	(29.2-36.3)
High education	48.9	(43.5-54.3)	High education	28.6	(25.2-32.2)
**Total (women and men)**	**17.9**	**(17.3-18.5)**	**Total (women and men)**	**17.9**	**(17.3-18.5)**

CI=Confidence interval
